# Systemic effects of type 2 diabetes therapies: an integrated perspective on the cardio–renal– cerebral–metabolic axis

**DOI:** 10.3389/fmed.2026.1746457

**Published:** 2026-03-24

**Authors:** Lin Zhu, Chen Chen, Feng Lu, Bingying Li

**Affiliations:** 1 University Town Hospital, Affiliated Hospital of Shandong University of Traditional Chinese Medicine, Department of Endocrinology, Jinan, China; 2 Dong'e County People's Hospital, Department of Endocrinology, Liaocheng, China; 3 Shandong University of Traditional Chinese Medicine Affiliated Hospital, Department of Radiology, Jinan, China

**Keywords:** cardio–renal–cerebral–metabolic axis, chronic inflammation, organ protection, precision medicine, type 2 diabetes

## Abstract

Type 2 diabetes (T2D) has been treated as an underlying disease—hyperglycemia, but is instead a systemic disease—mediating the network of neural, endocrine, and immune signaling. In recent years, the concept of the cardio–renal–cerebral–metabolic axis has provided an integrative pathophysiological framework for understanding the multisystem complications of diabetes. From this perspective, the present review systematically elucidates the substantial evolution in modern T2D therapeutic strategies from simple glycemic control to comprehensive multi-organ protection. The primary pathology is that high insulin resistance and chronic metabolic disturbances trigger oxidative stress and inflammation, which in turn drive a vicious cycle in the heart, kidneys, and brain. In this review, we demonstrate that new drugs based on sodium-glucose cotransporter 2 (SGLT2) inhibitors, glucagon-like peptide-1 (GLP-1) receptor agonists, and mineralocorticoid receptor antagonists, which are capable of lowering glucose to promote efficient glycemic control, decrease cardiovascular events, lower the risk of renal disease, and demonstrate neuroprotective properties as the key to organ protection. Additionally, non-pharmacological interventions and new treatments can be combined as a multi-targeting, multilayer management system. Furthermore, greater knowledge and integration of the cardio-renal-cerebral-metabolic axis could signal a shift toward precision medicine to stabilize the network's homeostasis and improve long-term patient outcomes.

## 1 Introduction

### 1.1 Overview of type 2 diabetes

A significant characteristic of type 2 diabetes is the interaction of insulin resistance with beta-cell dysfunction (1, 2). We start with impaired glucose uptake as peripheral tissue becomes less sensitive to glucose, and gradually decrease the effectiveness of the bio-cell compensatory function (3–6). Chronic inflammation and oxidative stress due to metabolic fluctuations are key links, including mitochondrial dysfunction, which leads to multi-organ injury (1, 4).

T2D is a worldwide health burden for 90%−95% of all diabetes cases and tens of millions of people worldwide. It remains one of the leading causes of death in low-income countries and among younger people (7–12). A recent GBD study corroborates that the disability-adjusted life years (DALYs) for T2D increased from around 27.4% in 1990 to 2021 (13, 14). Low- and middle-income Countries (LMICs) have been heavily affected due to rapid urbanization and lifestyle changes (13). High body mass index (BMI) remains the major risk factor for 52.2% of T2D-related DALY in 2021, 24.3% higher than in 1990 (15). Disease burden increases among those under 20 years of age: there were 128.7 and 439.9% of people under 20 years old between 1990 and 2021 (16). T2DT and hyperglycemia in China resulted in 0.9 million deaths and 26.8 million DALYs in 2021 (three times those of 1990), and are expected to be 18.17% by 2050 (16). Taken together, these results underscore the acceleration and the more youthful global burden of T3D due to obesity, which remain largely unknown (15, 16).

This disease is systemic, and we conclude that T2D is not merely a disease of glucose regulation but one caused by genetic, environmental, and lifestyle factors that affect multiple organs, such as the heart, kidneys, and brain (17, 18). The severe consequences of T2D include severe multisystem problems: dyslipidemia-induced risk of heart attack and stroke, diabetic death from kidney failure, and neurological disorders that share pathological mechanisms with Alzheimer's disease and increase patient disability and death (19, 20).

In summary, the core pathophysiological process of T2D manifests as progressive metabolic dysregulation and represents a complex network disorder characterized by multisystem interactions (17). Despite the global spread of risk factors that aggravate the disease burden, there is still a lack of effective early predictive biomarkers and individualized therapeutic tools (9, 18). Traditional models view problems as one-to-one or only include dual-organ interactions (e.g., cardio-renal). However, they fail to capture the overall influence of metabolic drivers on the brain and feedback loops. In recent years, researchers have proposed the idea of a cardio-renal-cerebral-metabolic axis to describe the molecular and physiological mechanisms underlying the multisystem dysfunction associated with diabetes, as a model for implementing therapeutic plans. Understanding the physiological and pathological interactions of cardio-Renal-Cerebral-metabolic, therefore, is a prerequisite for developing global systemic approaches.

### 1.2 The cardio–renal–cerebral–metabolic axis: an integrative pathophysiological model

The cardio-renal-cerebral-metabolic axis describes a multi-system network of interactions among the heart, kidneys, brain, and metabolic system via neural, endocrine, and metabolic signals. For diabetes, it highlights that glycemic control, cardiorenal function, and cognitive abilities are tightly coupled: If any part of this system fails, it triggers cascading effects that result in the coordinated deterioration of multiple organs and ultimately worsen patients' outcomes (21–24).

The proposed framework is conceptually distinct from existing multi-organ models. While the traditional Cardio-Renal Syndrome primarily focuses on hemodynamic cross-talk, and the Gut-Brain Axis emphasizes satiety signaling, these frameworks often fail to capture the systemic nature of metabolic comorbidities (25–27). In contrast, the Cardio-Renal-Cerebral-Metabolic Axis integrates these systems by positioning “Metabolic Dysregulation”—specifically chronic inflammation and insulin resistance—as the central upstream driver connecting the heart, kidneys, and brain, rather than merely hemodynamic consequences (28, 29). Recent evidence underscores that traditional models overlook the profound neurocognitive impact, where metabolic abnormalities directly impair blood-brain barrier integrity and synaptic function, leading to a two- to four-fold increase in cognitive impairment risk independent of vascular events (30–32). Clinically, this distinction justifies a paradigm shift from reactive management of dual-organ failure to proactive, holistic protection that targets these shared metabolic roots.

Rather than competing dual-organ schemes, the new model allows the brain to serve as the regulator, regulating metabolic functioning as a daily system. This would require therapy from shielding individual organs to repairing the whole system. The metabolic issues of diabetes (insulin resistance, inflammation) pose a double challenge; they damage cardiorenal integrity and, through pathways (e.g., gut-brain axis), reach the hippocampus, and make us cognitively impaired (33–37). Conversely, deterioration in brain regulatory functions, such as impaired neural plasticity, can weaken patients' capacity for disease self-management, creating a vicious cycle that further disrupts metabolic homeostasis (38–41).

Diabetes-induced myocardial dysfunction and heart rate variability may have both a serious risk and an underlying cause, vascular dysfunction of the kidneys, which drives one another, and leads to a cycle of concurrent cardiorenal decline (42, 43). The kidneys regulate the metabolic system by maintaining fluid balance and excreting glucose. If the kidneys are injured, it can activate the hypothalamic–pituitary–adrenal axis, which can worsen systemic metabolic dysregulation (44, 45). In terms of the brain, gut microbiota and associated hormones regulate glucose and lipid metabolism and interact with cognition via vagal pathways and metabolic abnormalities, e.g., hyperglycemia, which affect hippocampal metabolites as markers of cognitive dysfunction (23, 46). The metabolic system is the primary mediator of inter-organ damage when glucose and lipid metabolism cause oxidative stress and inflammation (22, 46).

**Figure 1:**
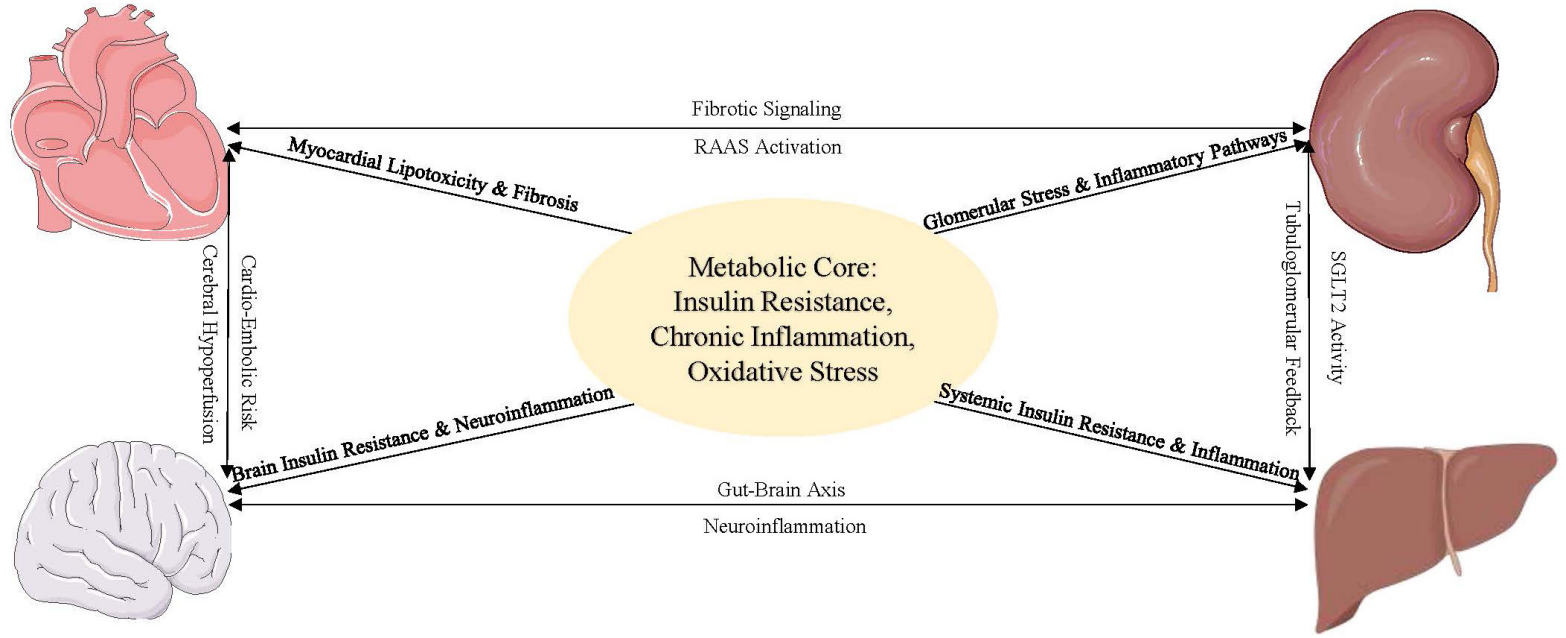
Interactive mechanisms of the cardio–renal–cerebral–metabolic axis.

Overall, the complexity of the cardio–renal–cerebral–metabolic axis underscores the need for integrated interventions that may provide a unified model of the multisystem complications of diabetes and a global perspective on this complex pathophysiological network. The essential mechanisms are summarized in Figure 1. The perspective for future treatment is to move from an individual-organization or signaling pathway approach to system-level approaches to restore network homeostasis (47, 48). Based on this systematic approach, the subsequent sections will discuss how therapies are evolving to target these nodes.

## 2 Major therapeutic strategies for type 2 diabetes

### 2.1 Pharmacological strategies: the evolution from glucose lowering to organ protection

Beyond the proposed switch to the cardio–renal–cerebral–metabolic axis, T2D has achieved a dramatic transition from simple glucose control to organ protection with therapeutic goals that no longer restricted to glycemic target, but toward systemic interventions that delay metabolic damage to heart, kidneys, and brain. This has been motivated by strong evidence in many large cardiovascular outcome trials, namely that certain new antidiabetic drugs can reduce the risks of heart failure, kidney disease progression and death while keeping glycemic control (26, 49). On this basis, the cardio–renal–cerebral–metabolic axis is based on the theory of such an integrated management strategy (50).

Among traditional medications, the United Kingdom Prospective Diabetes Study (UKPDS) proposed metformin as the first-line primary therapy because it improves the insulin sensitivity by activating the AMPK pathway (51). While older hypoglycemic agents can provide glucose lowering functions, they cannot offer long-term safety and organ-preserving benefits (52, 53). To clarify their role within the axis model, the individual organ effects and potential risks of the classical agents (Sulfonylureas, Thiazolidinediones, DPP-4 inhibitors, Insulin) are summarized in Table 1. Even though they work well in glycemic control, their unrepresentative cardiorenal benefit is far from being identical to new therapies. This highlights the inadequacy of a purely “glucose-centered” treatment model and motivates the development of novel agents with distinct organ mechanisms (54).

**Table 1:** Positioning of traditional glucose-lowering agents within the cardio–renal–cerebral–metabolic axis.

**Drug class**	**Cardiovascular impact**	**Renal impact**	**Neuro-cognitive impact**	**Metabolic profile and key risks**
Metformin	Neutral/potential benefit	Neutral	Potential benefit	Weight neutral
	Evidence suggests reduced risk of MI (UKPDS), though less robust than novel agents (e.g., SGLT2i/GLP-1 RA).	No direct nephroprotection beyond glycemic control; dose adjustment required based on eGFR.	Observational data link use to lower dementia risk; crosses the blood-brain barrier.	Risks: Vitamin B12 deficiency; lactic acidosis.
Sulfonylureas	Neutral	Neutral	Potential risk	Weight gain
	Cardiovascular safety is generally established, but lacks the specific protective benefits seen in newer classes.	No specific protective effects; requires dose adjustment in CKD.	Severe hypoglycemia events is strongly associated with increased risk of cognitive decline.	Risks: Severe hypoglycemia (dangerous for the elderly).
Thiazolidinediones	Mixed profile	Neutral	Potential benefit	Weight gain
	Reduces risk of stroke and MI, but carries a high risk of fluid retention and hospitalization for heart failure.	No significant impact on CKD progression beyond glycemic control.	Reduced recurrent stroke risk; potential insulin-sensitizing effects in the brain.	Risks: Fluid retention, edema, bone fractures, heart failure.
DPP-4 inhibitors	Neutral	Neutral	Neutral	Weight neutral
	Proven cardiovascular safety but no reduction in MACE or heart failure risks.	Safe in renal insufficiency; may significantly reduce albuminuria levels.	No consistent evidence of neuroprotection or cognitive harm.	Risks: Joint pain; Pancreatitis (rare).
Insulin	Neutral	Neutral	Indirect risk	Weight gain
	Effective for glycemic control; neutral for cardiovascular outcomes (ORIGIN trial).	Safe in advanced CKD; essential for preventing microvascular damage via glucose control.	Peripheral hyperinsulinemia does not equal central effect; hypoglycemia poses direct cognitive risks.	Risks: Hypoglycemia; Injection site reactions.

CV, cardiovascular; BBB, blood-brain barrier; CKD, chronic kidney disease; HF, heart failure; MI, myocardial infarction; MACE, major adverse cardiovascular events.

The new therapeutic approach has been demonstrated by recent studies conducted between 2023 and 2025. The FLOW trial provided the study on GLP-1 receptor agonists, showing that semaglutide reduced the risk of major kidney disease events by 22% (HR: 0.78) in CKD patients, irrespective of baseline use (55, 56). In terms of cardiovascular and metabolic integration, the SELECT study showed that semglutone reduced major adverse cardiovascular events (MACE) by 20% in overweight and obese patients while even without diabetes, suggesting the importance of targeting upstream metabolic drivers (57, 58). EMPEROR-Preserved and DELIVER presented evidence of the role of SGLT2 inhibitor in heart failure with preserving ejection fraction (HFpEF) in which there are still significant reductions in cardiovascular death or heart failure hospitalization by about 21% (59, 60). Finally, recent dual GIP/GLP-1 receptor agonist SURPASS showed superior potential, suggesting 27% reduction in MACE and significant improvement in renal endpoints compared to insulin glargine (61, 62). All these studies corroborated a shift toward a systematic cardio-renal-cerebral-metabolic management model.

This new generation sodium–glucose cotransporter 2 (SGLT2) inhibitors and glucagon-like peptide-1 (GLP-1) receptor agonists are the key players in interventions targeting the cardio–renal–cerebral–metabolic regime. The key components of their development, and most importantly their physiological functions, are their molecular functions. The SGLT 2 inhibitors repress the glucose and sodium uptake in the proximal tubule, inducing osmotic diuretic respiration and natriuresis. This facilitates the suppression of blood glucose, decreases the heart preload/afterload, and shifts the metabolic substrate consumption toward ketones (63–65). GLP-1 receptors suppress the secretion of insulin, slow down gastric digestion and reduce appetite to bring heavy losses and have anti-inflammatory and anti-atherosclerotic effects of improving the endothelial function and oxidative stress markers (66, 67).

**Figure 2:**
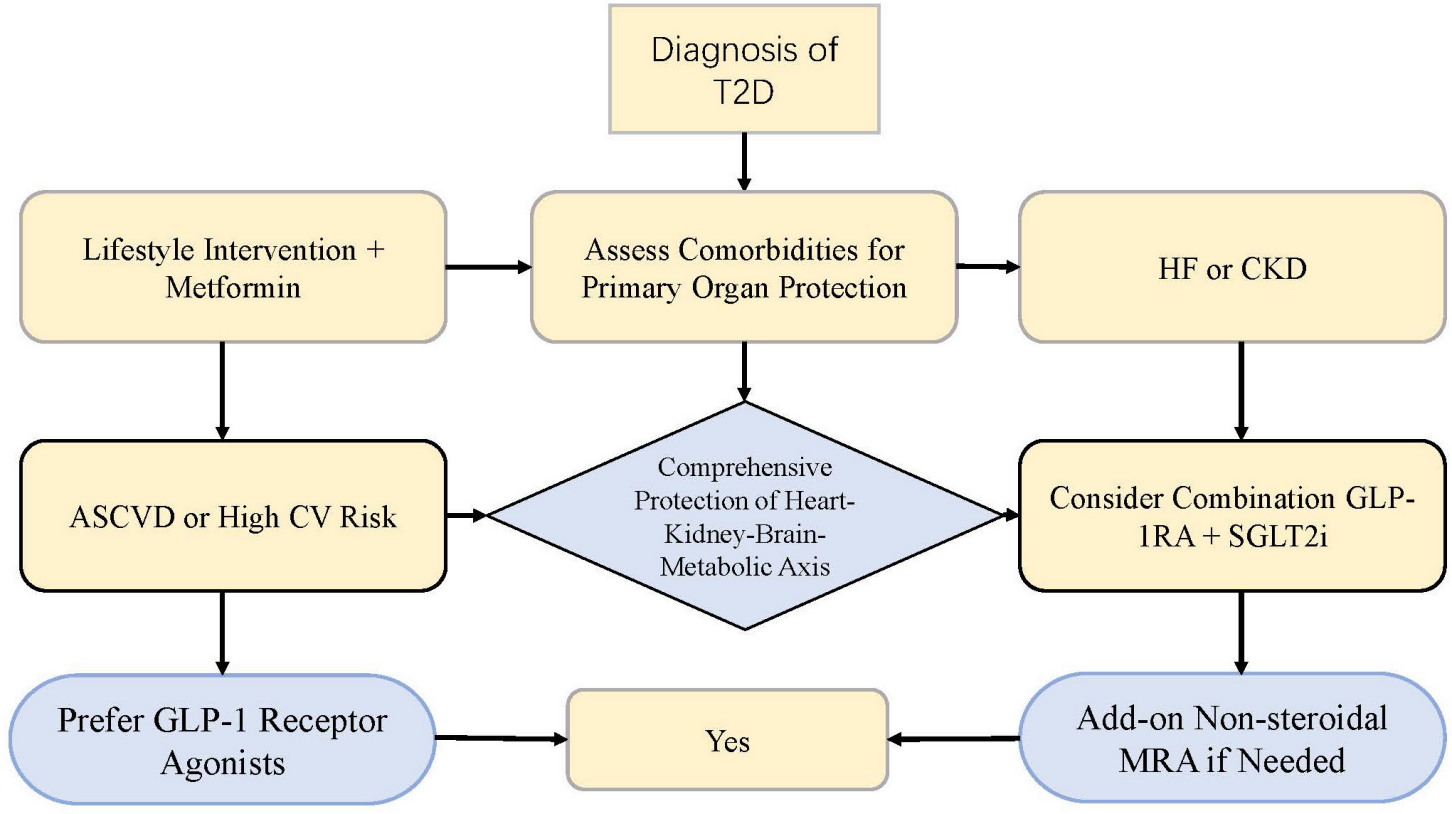
Modern therapeutic pathway for type 2 diabetes based on the cardio–renal–cerebral–metabolic axis.

Based on these mechanisms, modern T2D management has become a structured mechanism as illustrated in Figure 2. It starts with T2D diagnosis, and lifestyle change plus metformin is initiated. One can take into account comorbidities to decide which organ-protective drugs should be used: for atherosclerotic cardiovascular disease (ASCVD), or high cardiovascular risk, GLP-1 receptor agonists should be preferred; for heart failure (HF) or chronic kidney disease (CKD), SGLT2 inhibitors should be recommended. For patients with complex diseases, or a greater level of protection, GLP-1 receptor antagonists should be considered as a combination of GLP-1 receptor inhibitors and SGLTP2 inhibitors to obtain their enhanced cardiorenal benefits. In patients with high risk of disease such as T2D and CKD, a nonsteroidal mineralocorticoid receptor antagonist may also provide additional cardioreginal benefits. Overall, modern T2D treatment for T2 is composed by SGLP2 inhibitors, GLP-1 receptor antagonist and mineralocorticoid receptor antagonist (MRA) to go from “glucose lowering alone” to “systemic multi-organ protection and comorbity-based precision treatment pathways” (26, 68). However, pharmacological therapy is insufficient to address the metabolic dysregulation completely and including non-pharmacological and emerging therapies has been one of the key approaches to T2D management.

### 2.2 Non-pharmacological therapy

Non-pharmacological measures in controlling T2D in general work in synergy with the pharmacological measures to form the basis of disease management. Dietary and exercise measure insulin sensitivity, inflammatory response, and influence gut microbiota–based metabolites to allow for a long-term success of the clinical treatment from the metabolic point of view. Diet control: Healthy eating patterns such as high-fiber or Mediterranean diets can significantly improve glycemic homeostasis and reduce cardiovascular risk (69–71). Exercise not only maintains glycemic stability, but it is also effective against microvascular complications as an physiological anti-inflammatory strategy to maximize metabolic function (72, 73). Weight management is of primary use for obesity patients: evidence based weight reduction can be beneficial in terms of metabolic control; paired with structured lifestyle interventions, it might even result in disease recovery (74, 75). Furthermore, we demonstrated that changing the disease process could yield promising results in recent studies. For example, the DiRECT study showed that intensive weight control in primary care could lead to remission of 46% of patients within 1 year, which strongly rejects the idea of T2D as a gradual disease (76–78). Regarding long-term morbidity, though Look AHEAD found that lifestyle intervention may not be a major success in terms of major cardiovascular events, it clearly has multiple benefits for patients' survival. For example, this intervention was effective in secondary outcomes, including preventing obstructive sleep apnea and improving physical activity, which reinforces the importance of non-pharmacological treatments for individual patients (78–80). In that case, systematic non-pharmacological treatments can be clinically useful to slow down disease development and to make long term effects (81).

### 2.3 Emerging therapies

While cell treatments for T2D have been investigated, other cell- and gene-based therapies have promising promising future therapeutic potentials for stabilizing the cardio-renal-cerebral-metabolic axis. With cell therapy, mesenchymal stem cell (MSC) transplantation (due to its multi-directional differentiation and unique immunomodulatory features) could potentially promote pancreatic β-cell regeneration and repair damaged islet tissue (82, 83). Recent evidence highlights their systemic value beyond the pancreas: MSC-derived exosomes can deliver miRNAs to inhibit fibrosis and inflammation in the kidneys and heart, and potentially exert neuroprotective effects by modulating neuroinflammation, offering a “multi-organ repair” mechanism (84–86). Gene therapy aims at correcting metabolic pathway abnormalities at the root of the disease. Applications like the CRISPR/Cas9 genome-editing systems and GLP-1 gene delivery vectors have opened new opportunities for precise control of pathogenic genes (87–91). Specifically, these approaches can ensure continuous endogenous GLP-1 production, providing stable metabolic homeostasis superior to the fluctuations seen with daily pharmacotherapy (82, 92).

These new technologies have safety challenges, such as the impaired survival of MSCs in high-glucose environments and the immunogenicity of viral vectors (89, 93). Long-term stability, and ethical control remain prerequisites, but these innovations have provided a foundation for the future of systemic diabetes treatment (87, 94).

## 3 Effects of type 2 diabetes treatment on the heart

### 3.1 Association between diabetes and cardiovascular disease

Studies show that T2D is also an independent risk factor for heart disease. Large cohort data show that patients with T2d risk both coronary artery disease and heart failure, with patients being over four times more likely to develop coronary disease than non-diabetic patients (95, 96). Remarkably, even without overt coronary artery disease, T2D itself can also result in structural and functional abnormalities of the myocardium, a different clinical type that is called diabetic cardiomyopathy (97–99).

The key mechanisms of pathogenesis include multiple damages due to chronic hyperglycemia, insulin resistance and lipid metabolism: metabolic damage drives atherosclerosis via oxidative stress and endothelial disorders (100, 101); chronic inflammation, accumulation of advanced glycation end products (AGEs), and myocardial energy metabolic remodeling—characterized by impaired glucose utilization and excessive fatty acid oxidation—directly damage cardiomyocytes and induce fibrosis and apoptosis (102, 103); concurrently, characteristic coronary microcirculatory dysfunction further exacerbates myocardial ischemia and contractile impairment (104). Collectively, these factors lead to diabetic cardiomyopathy, which is characterized by early diastolic dysfunction and slow systolic degradation which becomes apparent even in absence of coronary artery lesions (105, 106). This condition carries critical clinical significance.

### 3.2 Cardioprotective effects and mechanisms of type 2 diabetes therapeutic agents

T2D patients are more than two times more likely to die from atherosclerotic death from heart disease than non-diabetic patients (100, 107, 108). Diabetes myocardial changes: metabolic defects, microcirculatory dysfunction and interstitial fibrosis, which all lead to progressive changes in the diastolic and systolic function (109). In order to track these disorders, based on the systemic strategies introduced in Section 2.1, clinical trials show significant organ-related benefits. For SGLT-2i, large-scale cardiovascular outcome trials (CVOTs) have always proven effective in reducing heart failure risk. Recent updates from EMPEROR-Preserved and DELIVER have expanded this benefit to patients with HFpEF, reducing the composite risk of cardiovascular death or hospitalization by 21% (59). The data show that SGLt-2si reduces mortality of the heart by about 30% and heart failure hospitalization by 35%, respectively. Results show SGLT-2i can reduce cardiovascular death by around 30% and heart failure hospitalization by 35% (50, 109–111). All the benefits of heart-specific hemodynamic optimization include reducing the stress of the ventricular wall and preventing the heart-frosis (112). On the other hand, glucagon-like peptide-1 receptor agonists (GLP-1 RAs) are more favorable for major adverse cardiovascular events by 12%−14% and non-fatal stroke (50, 109). These protective factors rely primarily on anti-atherosclerotic actions, such as plaque stabilization and improvements of coronary microvascular functioning (66). Nonsteroidal MRAs such as finerenone block mineralocorticoid receptors, reducing myocardial inflammation and fibrosis, and slowing down ventricular hypertrophy and diastolic dysfunction (50, 113). Collectively, such agents are central to cardioprotection, with SGLT-2i focusing on heart failure and GLP-1 targeting ischemic events. Effects of Type 2 Diabetes Treatment on the Kidneys.

### 3.3 Pathophysiological mechanisms of diabetic kidney disease

The main mechanism underlying diabetic kidney disease (DKD) in T2D stems from chronic hyperglycemia-induced metabolic dysregulation and multiple signaling abnormalities. Hyperglycemics promote increasing production of higher glycation end products (AGEs), activate receptor AGEs and produce more than abundant mitochondrial ROS production, directly damaging glomerular endothelial cells, podocytes and tubular epithelial cells (114–116). Glomerular remodeling and impaired tubuloglomerular feedback, leading to intraglomerular hypertension and hyperfiltration of kidney tissues, and a persistent increase in kidney function. In addition, the hyperglycemic environment keeps increasing and increasing the proliferation of various profibrotic and proinflammatory pathways: TGF-β promotes extracellular matrix accumulation and glomerulosclerosis and tubulointerstitial fibrosis; PKC promotes ROS production by NADPH oxidase activation and oxidative damage; NF-κB promotes proinflammatory cytokine releases, such as TNF-α and IL-1β, and cooperating with the NLRP3 inflammasome, which leads to local renal inflammation (114, 117–120). These interconnected processes form a vicious cycle of “oxidative stress–inflammation–fibrosis,” which constitutes the central mechanism underlying the progression of DKD (119, 121). Notably, renal impairment further activates neuroendocrine feedback systems—particularly the renin–angiotensin–aldosterone system (RAAS)—thereby aggravating cardiac load and contributing to the “cardio–renal vicious cycle.”

### 3.4 Renoprotective effects and mechanisms of type 2 diabetes therapeutic agents

As one of the most severe microvascular complications of T2D, diabetic kidney disease is a major cause of end-stage renal disease (ESRD) (122, 123). The novel drugs in turn can translate these systemic drugs into renal benefits, which result in a great success in delaying disease progression. For SGLT-2i, the primary renal-related benefit is from restoring tubuloglomerular feedback, which decreases intraglomerular pressure and filtration (124). Clinical trials have established that SGLT-2i greatly slows down estimated glomerular filtration rate (eGFR) and decreases the risk of ESRD with no glycemic control (122, 125). For GLP-1 RAs there has been evidence that the urinary albumin-to-creatinine ratio (UACR) and glomerular permeability were greatly reduced due to local anti-inflammatory processes and endothelial integrity (111). In addition nonsteroidal MRAs (e.g., finerenone) were found to slow down CKD progression and decrease ESRD incidence by suppressing inflammation and fibrosis within the renal interstitium (126). These results show a shift in nephrology from reactive management to proactive immunization of the kidney.

## 4 Effects of type 2 diabetes treatment on the kidneys

### 4.1 Type 2 diabetes and cognitive impairment: from metabolic dysregulation to brain injury

The epidemiological evidence for T2D is that it is a risk factor for cognitive decline, vascular dementia, and Alzheimer's disease. The pathogenesis is far from only metabolic and neurodegenerative features such as insulin signaling dysregulation, oxidative stress, and neuroinflammation. There are common “metabolic–neuronal injury” processes in the literature (127). At the medical level, several processes play important roles: neuronal insulin resistance leads to poor glucose use, leading to impaired synaptic plasticity of major regions such as hippocampus. *In vivo*, abnormal hippocampus N-acetylaspartate/creatine (NAA/Cr) ratios are observed in T2D patients, providing *in vivo* evidence of neuronal metabolic dysfunction and apoptosis. In parallel, persistent hyperglycemia leads to microvascular endothelial dysfunction and high BBB permeability by creating white matter lesions and localized ischemia due to small extracellular vesicle-induced vascular injury. Chronic inflammation and oxidative stress are also contributing to inflammation and reactive oxygen species (ROS) activation by triggering microglia triggering a neuroinflammatory process affecting BBB integrity (128, 129). These processes, coupled with inflammation and oxidative stress, are critical factors of neuronal injury in the cardio–renal–cerebral network.

Recent studies show that the level of cognitive impairment positively correlates with diabetes duration, glycemic variability and insulin resistance (130, 131). In general, our results indicate that T2D-associated cognitive dysfunction can be interpreted as a multi-target injury of the “vascular–neuronal–glial network” due to metabolic disruption.

### 4.2 Neuroprotective potential of type 2 diabetes therapeutic agents

In recent years, novel antidiabetic agents such as GLP-1 RAs have demonstrated significant neuroprotective potential beyond glycemic control (132, 133). Mechanically, such benefits are mediated by direct and indirect processes. For direct central processes, it has been previously established that GLP-1 RAs may enter the blood-brain barrier to activate certain receptors widely expressed in the hippocampus and cortex, stimulate BBDNF expression and improves synaptic plasticity, and inhibiting microglial activation to reduce brain inflammation and reduce Alzheimer's pathology (e.g., β-amyloid deposition and Tau hyperphosphorylation). For indirect systemic mechanisms, these agents protect the brain by optimizing glycemic and lipid control, reducing systemic chronic inflammation and oxidative stress, and promoting endothelial functioning to prevent neurovascular injury and maintain blood-front barrier integrity (134, 135). The clinical results show that GLP-1 RAs, such as liraglutide, semagluta, improve cognitive function in Alzheimer's disease animal models and prevent dopaminergic neuronal degeneration in Parkinson's disease models (134, 136). However, it is strictly necessary to differentiate that animal models are supported by the regenerative effects of the regenerations, however human evidence is mostly supported by secondary studies of cardiovascular outcome trials and observational studies. Further randomized controlled trials, in particular, of cognitive outcomes, are still ongoing and necessary to confirm these neuroprotective effects.

SGLT-2i indirectly helps to maintain brain function through peripheral mechanisms through lowering blood glucose, reducing inflammation and oxidative stress, and improving heart, kidney and brain perfusion for a good system of the central nervous system (137). Observational studies indicate that SGLT-2i use is associated with a lower risk of Alzheimer's disease (HR ≤ 0.67) and slower cognitive decline, potentially related to reduced cardiovascular events (50, 109). Note that this neuroprotective effect of SGLT-2i does not provide evidence for the central mechanisms due mainly to the optimisation of systemic metabolic status. Importantly, current clinical data of S GLT-2i are observational, and significant evidence from cognitive-related RCTs are sparse. In general, GLP-1 RAs and SGLDT-2 are promising but there remains a major gap between current evidence from CVOTs and current neurocognitive data, which is based on observational data. For these real-world data, however, certain biases, such as the “healthy user effect” or reverse causality, are dominant. Therefore, these agents are currently a “neuroprotective option” than existing cognitive therapy and must be confirmed through dedicated RCT with the main cognitive endpoints (138, 139).

## 5 Regulation of metabolism by type 2 diabetes treatment

### 5.1 Metabolic dysregulation: the common upstream driver of multi-organ injury

T2D is an intrinsically multibiotrial disease. The fundamental pathology such as insulin resistance, lipid metabolic abnormality and hyperuricemia not only occur, but also share common pathogenic mechanisms that cause severe losses in the heart, kidneys, and brain (140, 141). In T2D patients, skeletal muscle and fat tissue are affected by high insulin signaling, lipid metabolism issues include high triglycerides and central obesity, as metabolic dysfunction associated steatotic liver disease (MASLD) and hyperuricemia contribute to this imbalance (28, 142–145).

These metabolic abnormalities cause systemic chronic inflammation, oxidative stress and endothelial dysfunction and are responsible for multi-organ functional decline: chronic low-grade inflammation and excessive ROS production caused by adipose tissue dysfunction directly affect vascular endothelium, kidneys, and neurons; insulin resistance coupled with hyperglycemia induce endothelial dysfunctions and microcirculatory dysfunction, which are early symptoms of atherosclerosis, nephropathy and neurodegeneration; ectopic lipid deposition causes mitochondrial dysfunction and endoplasmic reticulum stress, further impairing multi-Organ cellular function (146–152). These pathological phenomena are a leading element of the cardio–renal–cerebral–metabolic axis; for instance, MASLD can also promote liver injury, heart failure, and cognitive decline through shared insulin resistance and inflammation with T2D and cardiovascular disease (29, 153–156). Thus, metabolic dysregulation is a co-morbidity, and the main source of cross-organ injury. Appropriate metabolic responses such as insulin sensitivity, lipid disorders and inflammation and oxidative stress are essential for jointly protecting the heart, kidneys, and brain (28, 148, 155).

### 5.2 Metabolic modulation by therapeutic agents

T2D's treatment goals have become more general than glycemic control to control systemic metabolic dysregulation, to improve glucose and lipid metabolism, insulin resistance and chronic inflammation in order to avoid cardiovascular and renal problems (157, 158). Metformin is a crucial ingredient in metabolic therapy. Metformin activates AMPK signaling to inhibit hepatic gluconeogenesis, increase peripheral insulin tolerance, and achieve mild weight loss and lipid control, which will be the basis of future multi-target metabolic management (159, 160).

To build upon this, GLP-1 RAs have large metabolic benefits: they can lower the weight by reducing the central appetite and slow gastric emptying, and lipid metabolism through lowering the serum triglycerides and high-density lipoprotein (22, 161). These agents alleviate hepatic steatosis by regulating gluconeogenesis and inflammation through adipose tissue–derived miRNAs, both enhancing insulin signaling (67). SGLT-2i achieve their own metabolic modulations by stimulating urinary glucose and sodium uptake, increasing fatty acid oxidation, moderately losing weight, and reducing systolic blood pressure and serum uric acid concentrations (161). Their metabolic benefits come from reducing their total energy burden, reduced visceral fat accumulation, and decreasing the proinflammatory cytokines (162). This multipathway metabolic remodeling provides an energetically useful system organ protection.

Together GLP-1 RAs and SGLT-2i have complementary metabolic effects: GLP 1 RAs enhance insulin production, SGLTs-2s enhance urinary glucose clearance, and they both have additive effects on glycemic and lipid control, while being conducive to cardio–renal stability (163, 164). Collectively, metformin, GLP-1 RAs and SGLT-2i combine to adjust glucose and lipid metabolism and energy homeostasis from “glycemic control alone” to “systemic metabolic remodeling” (158, 165).

### 5.3 Multifaceted effects of treatment strategies

Current T2D management strategies are evolving from single-target interventions to systematic integration of multi-pathway regimens. Evidence indicates that monotherapy is often insufficient to halt disease progression; thus, regimens combining agents with complementary mechanisms—specifically SGLT-2i and GLP-1 RAs—have become mainstream for restructuring metabolic networks (164, 166). Biologically, this combination plays roles both in a two-fold way: GLP-1 RAs, besides glucose control, improve heart health and decrease renal risk through anti-atherosclerotic and central energy-balance mechanisms, SGLT-2i lower blood glucose and enhance hemodynamics via osmotic diuresis, which increases the heart load and indirectly prevents kidney function (149, 167). This mechanism, not only maximizes glycemic control, but also restores the stability of the cardio–renal–cerebral–metabolic axis with dual hemodynamic and metabolic modulation (168, 169).

Beyond the two drug combinations, new therapies are focusing on unimolecular multi-receptor agonists which are more closely complementary. These multi-pronged treatments reduce systemic inflammation, improve insulin secretion, and promote metabolic homeostasis. They dramatically reduce oxidative stress and adipose tissue dysfunction (162, 170). By turning from simple add-on approaches to molecularly adapted approaches, this systematized approach can serve as the basis for precision medicine, finding individualized risk sources in order to significantly improve clinical performance and risk of complications (171, 172).

## 6 Integration and perspectives of the cardio–renal–cerebral–metabolic axis

The cardio–renal–cerebral–metabolic axis acts as a co-regulatory network where pathological interactions between downstream drivers are carried out by common drivers rather than individual organ injury. A major driving factor is the adaptation of the neuro-endocrine response, where excessive sympathetic tone (through cAMP/PKA signaling) and RAAS/MR axis overactivation promote myocardial remodeling, glomerular hypertension, and vascular fibrosis (173–175). Together, these hemodynamic insults are accompanied by metabolic-inflammatory processes; the continuous activation of NLRP3 inflammasome and NF-κB processes gives rise to systemic proinflammatory cytokines and uremic toxins that degrade endothelial integrity in the heart, kidneys and blood–brain barrier (176, 177). This vicious cycle is sustained by aberrant gut–brain signaling, characterized by dysregulated incretin secretion and microbiota-derived metabolites, which further destabilizes systemic energy homeostasis (178–180). Thus, the axis is a universal entity in which the therapy of treatment depends on those converging metabolic centers.

In general, the TyG index and other other indicators (e.g., TyG-BMI) are solid surrogate measures of insulin resistance and might be useful for therapeutic decision-making (181–183). Rather than serving merely as prognostic indicators for specific organ outcomes, elevated TyG levels should act as a “metabolic red flag” to trigger upstream interventions. Mechanistically, insulin resistance—quantified by the TyG index—amplifies systemic inflammation and oxidative stress, which are key drivers of multi-organ injury (184). As a result, in patients with high TyG indexes, therapies targeting insulin sensitivity and metabolic inflammation could be beneficial than agents targeting only glucose. This marker-guided treatment can detect “metabolic-inflammatory” phenotypes early and thus a clinician may prioritize agents interrupting the downstream drivers of the cardio–renal–cerebral–metabolic vicious cycle (185).

Examining such multidimensional regulation mechanisms can also be used as a theoretical basis for designing integrated therapies as summarized in Table 2, highlighting multi-organ injury in diabetes and offering biologically available targets for cross-organ treatment.

**Table 2:** Protective effects of novel antidiabetic agents on the cardio–renal–cerebral–metabolic axis: key evidence and mechanistic features.

**Drug class**	**Cardiac protection**	**Renal protection**	**Neuroprotection and cognition**	**Metabolic and other benefits**
SGLT2 inhibitors	Effect: Significantly reduce risk of heart failure hospitalization; some agents lower cardiovascular mortality.	Effect: Slow eGFR decline, reduce proteinuria, delay end-stage kidney disease.	Effect: Potential indirect neuroprotection (Evidence limited to observational data)	Core metabolic benefits: Promote urinary glucose excretion, modest weight loss (2–3 kg), lower blood pressure, reduce serum uric acid.
	Key evidence (HR, 95% CI): - HF hospitalization: 0.65 [0.54–0.79] (DAPA-HF) - Cardiovascular death (empagliflozin): 0.62 [0.49–0.77] (EMPA-REG OUTCOME)	Key evidence: - Renal composite endpoint (Dapagliflozin): 0.56 [0.45–0.68] (DAPA-CKD) - Significant reduction in UACR	Mechanisms: Improve systemic metabolism, reduce systemic inflammation and oxidative stress, stabilize cardiorenal function to optimize cerebral perfusion.	Unique action: Promote ketone utilization, optimize myocardial energy supply.
GLP-1 receptor agonists	Effect: Reduce major adverse cardiovascular events, particularly protective against stroke.	Effect: Lower risk of new or worsening kidney disease, significantly reduce proteinuria.	Effect: Direct neuroprotective potential, reduce dementia risk.	Core metabolic benefits: Robust weight loss (5%−10%), improved lipid profile, significant reduction of liver fat content.
	Key evidence (HR, 95% CI): - MACE: 0.86–0.88 (LEADER, SUSTAIN-6) - Nonfatal stroke (semaglutide): 0.61 [0.38–0.99]	Key evidence: - New-onset macroalbuminuria: 0.70–0.80 (LEADER Renal analysis) - Significant reduction in UACR	Mechanisms: Activation of central GLP-1 receptors, enhanced synaptic plasticity, reduced neuroinflammation, decreased Aβ deposition.	Unique action: Central appetite suppression.
Non-steroidal MRA	Effect: Reduce cardiovascular composite endpoints (MI, stroke, HF hospitalization, CV death).	Effect: Significantly slow progression of chronic kidney disease, lower risk of end-stage kidney disease.	Effect: Evidence insufficient.	Core metabolic benefits: Cardiovascular and renal protection independent of glycemic and blood pressure effects.
	Key evidence (HR, 95% CI): - CV composite endpoint: 0.86 [0.78–0.95]	Key evidence: - Renal composite endpoint: 0.82 [0.73–0.93] - Sustained ~30% reduction in UACR	Mechanisms/Implications: Systemic anti-inflammatory effects may indirectly benefit neuroinflammation; improved kidney function may reduce uremic toxin–induced brain damage.	Role: “Add-on” agent targeting cardiorenal outcomes, does not directly lower glucose.

HR, hazard ratio; CI, confidence interval; eGFR, estimated glomerular filtration rate; UACR, urine albumin-to-creatinine ratio; MACE, major adverse cardiovascular events; CKD, chronic kidney disease; T2D, type 2 diabetes.

## 7 Conclusion

The therapeutic paradigm of T2D has undergone a major evolution from traditional glycemic control to multi-system integrated regulation centered on the cardio–renal–cerebral–metabolic axis. This conceptual update stems from redefining the disease: T2D is not merely a metabolic disorder driven by insulin resistance and β-cell dysfunction but a systemic disease interconnected through neural, endocrine, and immune signaling networks. Modern therapeutic strategies that synergistically modulate the heart, kidney, brain, and metabolic systems signify a transition from “glucose-centric” management to “multi-organ protection,” substantially improving overall patient prognosis.

In clinical practice, SGLT2i, GLP-1 RAs, and ns-MRAs form the backbone of organ-protective therapy, offering multiple benefits beyond glycemic control. These pharmacologic interventions, combined with dietary modifications, exercise therapy, and other non-pharmacological approaches, act through shared mechanisms such as improving insulin sensitivity and reducing chronic inflammation to establish a multi-target, multi-level management framework. Additionally, emerging cell and gene therapies, despite safety challenges, provide new avenues for fundamentally reversing disease pathology.

Future clinical application of the cardio–renal–cerebral–metabolic framework requires a transition from generalized guidelines to phenotype-driven precision medicine. Specifically, therapeutic stratification should be guided by a multi-dimensional assessment of available biomarkers. Clinical trajectories defined by eGFR and UACR levels should dictate the early initiation of SGLT-2i and nonsteroidal MRAs to prevent cardiorenal progression. For patients exhibiting metabolic-inflammatory phenotypes, TyG-related indices and MASLD markers such as FIB-4 can serve as indicators to prioritize GLP-1 RAs, leveraging their potent anti-inflammatory and antifibrotic effects. Regarding cognitive preservation, baseline cognitive metrics like the MMSE combined with neuroimaging markers are essential to identify at-risk patients who may benefit most from the neuroprotective potential of GLP-1 RAs. Pursuing these directions will facilitate the transition from broad-spectrum management to high-precision, personalized care.

## References

[1] Han T Ko E Kim M Choi M Lee C Kim I-H . Mori ramulus inhibits pancreatic β-cell apoptosis and prevents insulin resistance by restoring hepatic mitochondrial function. Antioxidants. (2021) 10:901. doi: 10.3390/antiox10060901 34204891 PMC8229938

[2] Arora A Behl T Sehgal A Singh S Sharma N Bhatia S . Unravelling the involvement of gut microbiota in type 2 diabetes mellitus. Life Sci. (2021) 273:119311. doi: 10.1016/j.lfs.2021.119311 33662428

[3] Prasad MK Mohandas S Ramkumar KM. Dysfunctions, molecular mechanisms, and therapeutic strategies of pancreatic β-cells in diabetes. Apoptosis. (2023) 28:958–76. doi: 10.1007/s10495-023-01854-0 37273039

[4] Yong J Johnson JD Arvan P Han J Kaufman RJ. Therapeutic opportunities for pancreatic β-cell Er stress in diabetes mellitus. Nat Rev Endocrinol. (2021) 17:455–67. doi: 10.1038/s41574-021-00510-4 34163039 PMC8765009

[5] Kishima H Mine T Fukuhara E Kitagaki R Asakura M Ishihara M. Efficacy of sodium-glucose cotransporter 2 inhibitors on outcomes after catheter ablation for atrial fibrillation. Clin Electrophysiol. (2022) 8:1393–404. doi: 10.1016/j.jacep.2022.08.004 36424008

[6] Shum M Segawa M Gharakhanian R Viñuela A Wortham M Baghdasarian S . Deletion of Abcb10 in beta-cells protects from high-fat diet induced insulin resistance. Mol Metab. (2022) 55:101403. doi: 10.1016/j.molmet.2021.101403 34823065 PMC8689243

[7] Gupta P Taiyab A Hassan MI. Emerging role of protein kinases in diabetes mellitus: from mechanism to therapy. Adv Protein Chem Struct Biol. (2021) 124:47–85. doi: 10.1016/bs.apcsb.2020.11.001 33632470

[8] Peñalvo JL. The impact of taxing sugar-sweetened beverages on diabetes: a critical review. Diabetologia. (2024) 67:420–9. doi: 10.1007/s00125-023-06064-6 38177563

[9] Wang M Yang Q Li Y Zhao Y Zou J Luan F . Therapeutic potential of traditional chinese medicine and mechanisms for the treatment of type 2 diabetes mellitus. Chin Med. (2025) 20:157. doi: 10.1186/s13020-025-01222-x 41044789 PMC12495626

[10] Vaidya AR Wolska N Vara D Mailer RK Schröder K Pula G. Diabetes and thrombosis: a central role for vascular oxidative stress. Antioxidants. (2021) 10:706. doi: 10.3390/antiox10050706 33946846 PMC8146432

[11] Lam BQ Srivastava R Morvant J Shankar S Srivastava RK. Association of diabetes mellitus and alcohol abuse with cancer: molecular mechanisms and clinical significance. Cells. (2021) 10:3077. doi: 10.3390/cells10113077 34831299 PMC8620339

[12] Perng W Conway R Mayer-Davis E Dabelea D. Youth-onset type 2 diabetes: the epidemiology of an awakening epidemic. Diabetes Care. (2023) 46:490–9. doi: 10.2337/dci22-0046 36812420 PMC10090267

[13] Liu J Bai R Chai Z Cooper ME Zimmet PZ Zhang L. Low-and middle-income countries demonstrate rapid growth of type 2 diabetes: an analysis based on global burden of disease 1990–2019 data. Diabetologia. (2022) 65:1339–52. doi: 10.1007/s00125-022-05713-6 35587275 PMC9118183

[14] Cousin E Schmidt MI Ong KL Lozano R Afshin A Abushouk AI . Burden of diabetes and hyperglycaemia in adults in the americas, 1990–2019: a systematic analysis for the global burden of disease study 2019. Lancet Diabetes Endocrinol. (2022) 10:655–67. doi: 10.1016/S2213-8587(22)00186-3 35850129 PMC9399220

[15] Ong KL Stafford LK McLaughlin SA Boyko EJ Vollset SE Smith AE . Global, regional, and national burden of diabetes from 1990 to 2021, with projections of prevalence to 2050: a systematic analysis for the global burden of disease study 2021. Lancet. (2023) 402:203–34. doi: 10.1016/S0140-6736(23)02044-5 37356446 PMC10364581

[16] Zhang H Jia Q Song P Li Y Jiang L Fu X . Incidence, prevalence, and burden of type 2 diabetes in China: trend and projection from 1990 to 2050. Chin Med J. (2025) 138:1447–55. doi: 10.1097/CM9.0000000000003536 40375461 PMC12180826

[17] Barbieri M Prattichizzo F La Grotta R Matacchione G Scisciola L Fontanella RA . Is it time to revise the fighting strategy toward type 2 diabetes? Sex and pollution as new risk factors. Ageing Res Rev. (2024) 99:102405. doi: 10.1016/j.arr.2024.102405 38971321

[18] Prasad RB Hakaste L Tuomi T. Clinical use of polygenic scores in type 2 diabetes: challenges and possibilities. Diabetologia. (2025) 68:1361–74. doi: 10.1007/s00125-025-06419-1 40186687 PMC12177005

[19] Tang X Yan X Zhou H Huang G Niu X Jiang H . Associations of insulin resistance and beta-cell function with abnormal lipid profile in newly diagnosed diabetes. Chin Med J. (2022) 135:2554–62. doi: 10.1097/CM9.0000000000002075 35245924 PMC9944004

[20] Kale MB Bhondge HM Wankhede NL Shende PV Thanekaer RP Aglawe MM . Navigating the intersection: diabetes and Alzheimer's intertwined relationship. Ageing Res Rev. (2024) 100:102415. doi: 10.1016/j.arr.2024.102415 39002642

[21] Argyrakopoulou G Gitsi E Dalamaga M Kokkinos A. Obesity and the gut-brain axis in type 1 diabetes mellitus: terra incognita? Curr Obes Rep. (2025) 14:1–11. doi: 10.1007/s13679-025-00654-8 40707821 PMC12289725

[22] Wat LW Svensson KJ. Novel secreted regulators of glucose and lipid metabolism in the development of metabolic diseases. Diabetologia. (2024) 67:2626–36. doi: 10.1007/s00125-024-06253-x 39180580 PMC12087937

[23] Biswas R Capuano AW Mehta RI Barnes LL Bennett DA Arvanitakis Z. Review of associations of diabetes and insulin resistance with brain health in three harmonised cohort studies of ageing and dementia. Diabetes Metab Res Rev. (2025) 41:e70032. doi: 10.1002/dmrr.70032 39873127 PMC11774135

[24] Marassi M Fadini GP. The cardio-renal-metabolic connection: a review of the evidence. Cardiovasc Diabetol. (2023) 22:195. doi: 10.1186/s12933-023-01937-x 37525273 PMC10391899

[25] Natale P Palmer SC Tunnicliffe DJ Toyama T Strippoli GF. Dipeptidyl peptidase 4 (Dpp-4) inhibitors for people with chronic kidney disease and diabetes. Cochrane Database Syst Rev. (2023) 2023:CD015906. doi: 10.1002/14651858.CD015906 41263251 PMC12631961

[26] Liu H Sridhar VS Boulet J Dharia A Khan A Lawler PR . Cardiorenal protection with Sglt2 inhibitors in patients with diabetes mellitus: from biomarkers to clinical outcomes in heart failure and diabetic kidney disease. Metabolism. (2022) 126:154918. doi: 10.1016/j.metabol.2021.154918 34699838

[27] Heidarianpour A Mohammadi F Keshvari M Mirazi N. Ameliorative effects of endurance training and matricaria chamomilla flowers hydroethanolic extract on cognitive deficit in type 2 diabetes rats. Biomed Pharmacother. (2021) 135:111230. doi: 10.1016/j.biopha.2021.111230 33434853

[28] Shah N Sanyal AJ. A pragmatic management approach for metabolic dysfunction-associated steatosis and steatohepatitis. Am J Gastroenterol. (2025) 120:75–82. doi: 10.14309/ajg.0000000000003215 39569874

[29] Rohm TV Meier DT Olefsky JM Donath MY. Inflammation in obesity, diabetes, and related disorders. Immunity. (2022) 55:31–55. doi: 10.1016/j.immuni.2021.12.013 35021057 PMC8773457

[30] Kim WJ Lee SJ Lee E Lee EY Han K. Risk of incident dementia according to glycemic status and comorbidities of hyperglycemia: a nationwide population-based cohort study. Diabetes Care. (2022) 45:134–41. doi: 10.2337/dc21-0957 34711638

[31] Abdelsaid K Abdul Y Jamil S Li W Ergul A. A novel vascular multi-etiology model of Alzheimer's disease-related dementias (Adrd). Alzheimers Dement. (2025) 21:e098657. doi: 10.1002/alz70855_098657

[32] Shen S Liao Q Wong YK Chen X Yang C Xu C . The role of melatonin in the treatment of type 2 diabetes mellitus and Alzheimer's disease. Int J Biol Sci. (2022) 18:983. doi: 10.7150/ijbs.66871 35173531 PMC8771831

[33] Shen X Zhao F Zhao Z Yu J Sun Z. Probiotics: a potential strategy for improving diabetes mellitus complicated with cognitive impairment. Microbiol Res. (2025) 290:127960. doi: 10.1016/j.micres.2024.127960 39515265

[34] Vargas-Soria M Garcia-Alloza M Corraliza-Gomez M. Effects of diabetes on microglial physiology: a systematic review of *in vitro*, preclinical and clinical studies. J Neuroinflammation. (2023) 20:57. doi: 10.1186/s12974-023-02740-x 36869375 PMC9983227

[35] Chen J Guo P Han M Chen K Qin J Yang F. Cognitive protection of sinomenine in type 2 diabetes mellitus through regulating the Egf/Nrf2/Ho-1 signaling, the microbiota-gut-brain axis, and hippocampal neuron ferroptosis. Phytother Res. (2023) 37:3323–41. doi: 10.1002/ptr.7807 37036428

[36] Ye X-x Jiang Q-y Wu M-j Ye Q-h Zheng H. Transplant of fecal microbiota from healthy young mice relieves cognitive defects in late-stage diabetic mice by reducing metabolic disorders and neuroinflammation. Acta Pharmacologica Sinica. (2024) 45:2513–26. doi: 10.1038/s41401-024-01340-6 38992120 PMC11579283

[37] Chen L Li Y Zhang X Du X Zhang Y Li X . Fucoidan prevents diabetic cognitive dysfunction via promoting Tet2-mediated active DNA demethylation in high-fat diet induced diabetic mice. Int J Biol Macromol. (2024) 278:134186. doi: 10.1016/j.ijbiomac.2024.134186 39173790

[38] Liu Y Ma X Xu J Wang X Liu L Ren X . Dietary n-3 polyunsaturated fatty acids intervention ameliorates cognitive dysfunction in db/db mice by mitigating cortical insulin resistance, mitochondrial dysfunction, and energy metabolism impairment J Adv Res. (2026) 81:437–51. doi: 10.1016/j.jare.2025.06.044 40541777 PMC12957818

[39] Baier MP Ranjit R Owen DB Wilson JL Stiles MA Masingale AM . Cellular senescence is a central driver of cognitive disparities in aging. Aging Cell. (2025) 24:e70041. doi: 10.1111/acel.70041 40077862 PMC12151884

[40] Xu H Chen H Li Y Luo T Zhao D Chen X . Dietary formaldehyde: a silent aggravator of diabetes and cognitive impairments. Nutr Diabetes. (2025) 15:35. doi: 10.1038/s41387-025-00390-x 40830331 PMC12365067

[41] Cui J Robert C Teh CM Jun Yi EC Chong JR Tan BY . Interactive effect of diabetes mellitus and subclinical Mri markers of cerebrovascular disease on cognitive decline and incident dementia: a memory-clinic study. Alzheimers Res Ther. (2024) 16:214. doi: 10.1186/s13195-024-01577-7 39363381 PMC11448036

[42] Strocchi M Hammersley DJ Halliday BP Prasad SK Niederer SA. Cardiac digital twins: a tool to investigate the function and treatment of the diabetic heart. Cardiovasc Diabetol. (2025) 24:293. doi: 10.1186/s12933-025-02839-w 40682127 PMC12275252

[43] Raleigh MJ Pasricha SV Nauth A Ward MR Connelly KA. Endothelial progenitor cells for diabetic cardiac and kidney disease. Stem Cells Transl Med. (2024) 13:625–36. doi: 10.1093/stcltm/szae025 38733609 PMC11227977

[44] Heather LC Gopal K Srnic N Ussher JR. Redefining diabetic cardiomyopathy: perturbations in substrate metabolism at the heart of its pathology. Diabetes. (2024) 73:659–70. doi: 10.2337/dbi23-0019 38387045 PMC11043056

[45] Faniyan TS Zhang X Morgan DA Robles J Bathina S Brookes PS . A kidney-hypothalamus axis promotes compensatory glucose production in response to glycosuria. Elife. (2024) 12:RP91540. doi: 10.7554/eLife.91540.4 39082939 PMC11290820

[46] Zhang L Li D Yi P Shi J Guo M Yin Q . Peripheral origin exosomal micrornas aggravate glymphatic system dysfunction in diabetic cognitive impairment. Acta Pharmaceutica Sinica B. (2023) 13:2817–25. doi: 10.1016/j.apsb.2023.03.018 37521866 PMC10372831

[47] Jeon JY Kim DJ. Benefit and safety of sodium-glucose co-transporter 2 inhibitors in older patients with type 2 diabetes mellitus. Diabetes Metab J. (2024) 48:837–46. doi: 10.4093/dmj.2024.0317 39313229 PMC11449826

[48] Pohlman N Patel PN Essien UR Tang JJ Joseph JJ. Novel cardiometabolic medications in the cardiovascular-kidney-metabolic syndrome era. J Clin Endocrinol Metab. (2025) 110:2105–22. doi: 10.1210/clinem/dgaf295 40388382 PMC12823194

[49] Khunti K Zaccardi F Amod A Aroda VR Aschner P Colagiuri S . Glycaemic control is still central in the hierarchy of priorities in type 2 diabetes management. Diabetologia. (2025) 68:17–28. doi: 10.1007/s00125-024-06254-w 39155282 PMC11663178

[50] Shi Q Nong K Vandvik PO Guyatt GH Schnell O Rydén L . Benefits and harms of drug treatment for type 2 diabetes: systematic review and network meta-analysis of randomised controlled trials. Bmj. (2023) 381:e074068. doi: 10.1136/bmj-2022-074068 37024129 PMC10077111

[51] Satin LS Soleimanpour SA Walker EM. New aspects of diabetes research and therapeutic development. Pharmacol Rev. (2021) 73:1001–15. doi: 10.1124/pharmrev.120.000160 34193595 PMC8274312

[52] Xiao J Li M Cai R Huang H Yu H Huang L . Smart pharmaceutical monitoring system with personalized medication schedules and self-management programs for patients with diabetes: development and evaluation study. J Med Internet Res. (2025) 27:e56737. doi: 10.2196/56737 39933171 PMC11862767

[53] Hoyt JA Cozzi E D'Alessio DA Thompson CC Aroda VR. A look at duodenal mucosal resurfacing: rationale for targeting the duodenum in type 2 diabetes. Diabetes Obes Metab. (2024) 26:2017–28. doi: 10.1111/dom.15533 38433708

[54] Johnson J Jaggers RM Gopalkrishna S Dahdah A Murphy AJ Hanssen NM . Oxidative stress in neutrophils: implications for diabetic cardiovascular complications. Antioxid Redox Signal. (2022) 36:652–66. doi: 10.1089/ars.2021.0116 34148367 PMC9057880

[55] Rossing P Bakris G Perkovic V Pratley R Tuttle KR Mahaffey KW . Effects of semaglutide with or without concomitant mineralocorticoid receptor antagonist use in participants with type 2 diabetes and chronic kidney disease: a flow trial prespecified secondary analysis. Diabetes Care. (2025) 48:1878–87. doi: 10.2337/figshare.29469482 40730031 PMC12583412

[56] Mahaffey KW Tuttle KR Arici M Baeres FM Bakris G Charytan DM . Cardiovascular outcomes with semaglutide by severity of chronic kidney disease in type 2 diabetes: the flow trial. Eur Heart J. (2025) 46:1096–108. doi: 10.1093/eurheartj/ehae613 39211948 PMC11931213

[57] Deanfield J Lincoff AM Kahn SE Emerson SS Lingvay I Scirica BM . Semaglutide and cardiovascular outcomes by baseline and changes in adiposity measurements: a prespecified analysis of the select trial. Lancet. (2025) 406:2257–68. doi: 10.1016/S0140-6736(25)01375-3 41138739

[58] Colhoun HM Lingvay I Brown PM Deanfield J Brown-Frandsen K Kahn SE . Long-term kidney outcomes of semaglutide in obesity and cardiovascular disease in the select trial. Nat Med. (2024) 30:2058–66. doi: 10.1038/s41591-024-03015-5 38796653 PMC11271413

[59] Bhatt DL Verma S Pitt B. Emperor-preserved: a promise fulfilled. Cell Metab. (2021) 33:2099–103. doi: 10.1016/j.cmet.2021.10.011 34731652

[60] Jhund PS Claggett BL Talebi A Butt JH Gasparyan SB Wei L-J . Effect of dapagliflozin on total heart failure events in patients with heart failure with mildly reduced or preserved ejection fraction: a prespecified analysis of the deliver trial. JAMA Cardiol. (2023) 8:554–63. doi: 10.1001/jamacardio.2023.0711 37099283 PMC10134044

[61] Wu J-Y Chen C-C Tu WL Hsu W-H Liu T-H Tsai Y-W . Clinical impact of tirzepatide on patients with Osa and obesity. Chest. (2025) 168:785–96. doi: 10.1016/j.chest.2025.03.030 40254150

[62] Apperloo EM Tuttle KR Pavo I Haupt A Taylor R Wiese RJ . Tirzepatide associated with reduced albuminuria in participants with type 2 diabetes: pooled *post hoc* analysis from the randomized active-and placebo-controlled surpass-1–5 clinical trials. Diabetes Care. (2025) 48:430–6. doi: 10.2337/dc24-1773 39746157 PMC11870291

[63] Panico C Bonora B Camera A Chilelli NC Prato GD Favacchio G . Pathophysiological basis of the cardiological benefits of Sglt-2 inhibitors: a narrative review. Cardiovasc Diabetol. (2023) 22:164. doi: 10.1186/s12933-023-01855-y 37391739 PMC10314539

[64] Lim VG He H Lachlan T Ng GA Kyrou I Randeva HS . Impact of sodium-glucose co-transporter inhibitors on cardiac autonomic function and mortality: no time to die. EP Europace. (2022) 24:1052–7. doi: 10.1093/europace/euab321 35080624

[65] Bugga P Mohammed SA Alam MJ Katare P Meghwani H Maulik SK . Empagliflozin prohibits high-fructose diet-induced cardiac dysfunction in rats via attenuation of mitochondria-driven oxidative stress. Life Sci. (2022) 307:120862. doi: 10.1016/j.lfs.2022.120862 35934058

[66] Rubio-Ruíz ME Plata-Corona JC Soria-Castro E Díaz-Juárez JA Sánchez-Aguilar M. Pleiotropic effects of peroxisome proliferator-activated receptor alpha and gamma agonists on myocardial damage: molecular mechanisms and clinical evidence—a narrative review. Cells. (2024) 13:1488. doi: 10.3390/cells13171488 39273057 PMC11394383

[67] Weng S-W Wu J-C Shen F-C Chang Y-H Su Y-J Lian W-S . Chaperonin counteracts diet-induced non-alcoholic fatty liver disease by aiding sirtuin 3 in the control of fatty acid oxidation. Diabetologia. (2023) 66:913–30. doi: 10.1007/s00125-023-05869-9 36692509

[68] Gómez-Huelgas R Sanz-Cánovas J Cobos-Palacios L López-Sampalo A Pérez-Belmonte LM. Glucagon-like peptide-1 receptor agonists and sodium– glucose cotransporter 2 inhibitors for cardiovascular and renal protection: a treatment approach far beyond their glucose-lowering effect. Eur J Intern Med. (2022) 96:26–33. doi: 10.1016/j.ejim.2021.11.008 34799233

[69] Colak H Larik GNF van Baak MA Canfora EE. Effects of isolated single fibers, fiber mixtures, and fiber-rich whole foods on glucose homeostasis in individuals with overweight and obesity: a systematic review and meta-analysis. Clin Nutr. (2025) 52:236–51. doi: 10.1016/j.clnu.2025.08.003 40803102

[70] Lotan R Ravona-Springer R Shakked J Lin H-M Ouyang Y Shahar DR . Greater intake of the medi diet is associated with better cognitive trajectory in older adults with type 2 diabetes. Diabetes Res Clin Pract. (2022) 190:109989. doi: 10.1016/j.diabres.2022.109989 35820563 PMC13195624

[71] Barrea L Verde L Colao A Mandarino LJ Muscogiuri G. Medical nutrition therapy for the management of type 2 diabetes mellitus. Nat Rev Endocrinol. (2025) 21:769–82. doi: 10.1038/s41574-025-01161-5 40817355

[72] Han S Wu Q Wang M Yang M Sun C Liang J . An integrative profiling of metabolome and transcriptome in the plasma and skeletal muscle following an exercise intervention in diet-induced obese mice. J Mol Cell Biol. (2023) 15:mjad016. doi: 10.1093/jmcb/mjad016 36882217 PMC10576543

[73] Garcia-Hermoso A Ramirez-Velez R Diez J Gonzalez A Izquierdo M. Exercise training-induced changes in exerkine concentrations may be relevant to the metabolic control of type 2 diabetes mellitus patients: a systematic review and meta-analysis of randomized controlled trials. J Sport Health Sci. (2023) 12:147–57. doi: 10.1016/j.jshs.2022.11.003 36351545 PMC10105032

[74] Naeh A Maor-Sagie E Hallak M Toledano Y Gabbay-Benziv R. Greater risk of type 2 diabetes progression in multifetal gestations with gestational diabetes: the impact of obesity. Am J Obstet Gynecol. (2024) 231:259.e1–10. doi: 10.1016/j.ajog.2023.11.1246 38360449

[75] McInnes N Hall S Lochnan HA Harris SB Punthakee Z Sigal RJ . Diabetes remission and relapse following an intensive metabolic intervention combining insulin glargine/lixisenatide, metformin and lifestyle approaches: results of a randomised controlled trial. Diabetes Obes Metab. (2023) 25:3347–55. doi: 10.1111/dom.15234 37580972

[76] Taylor R Al-Mrabeh A Sattar N. Understanding the mechanisms of reversal of type 2 diabetes. Lancet Diabetes Endocrinol. (2019) 7:726–36. doi: 10.1016/S2213-8587(19)30076-2 31097391

[77] Lean ME Leslie WS Barnes AC Brosnahan N Thom G McCombie L . Primary care-led weight management for remission of type 2 diabetes (direct): an open-label, cluster-randomised trial. Lancet. (2018) 391:541–51. doi: 10.1016/S0140-6736(17)33102-1 29221645

[78] Lean ME Leslie WS Barnes AC Brosnahan N Thom G McCombie L . Durability of a primary care-led weight-management intervention for remission of type 2 diabetes: 2-year results of the direct open-label, cluster-randomised trial. Lancet Diabetes Endocrinol. (2019) 7:344–55. doi: 10.1016/S2213-8587(19)30068-3 30852132

[79] Kuna ST Reboussin DM Strotmeyer ES Millman RP Zammit G Walkup MP . Effects of weight loss on obstructive sleep apnea severity. Ten-year results of the sleep ahead study. Am J Respir Crit Care Med. (2021) 203:221–9. doi: 10.1164/rccm.201912-2511OC 32721163 PMC7874414

[80] Houston DK Neiberg RH Miller ME Hill JO Jakicic JM Johnson KC . Physical function following a long-term lifestyle intervention among middle aged and older adults with type 2 diabetes: the look ahead study. J Gerontol Series A. (2018) 73:1552–9. doi: 10.1093/gerona/glx204 29053861 PMC6175031

[81] Benton JS Cotterill S Hawkes RE Miles LM French DP. Changes in a digital type 2 diabetes self-management intervention during national rollout: mixed methods study of fidelity. J Med Internet Res. (2022) 24:e39483. doi: 10.2196/39483 36476723 PMC9773035

[82] Shao Za Zhang X Cai J Lu F. Glucagon-like peptide-1: a new potential regulator for mesenchymal stem cells in the treatment of type 2 diabetes mellitus and its complication. Stem Cell Res Ther. (2025) 16:248. doi: 10.1186/s13287-025-04369-4 40390070 PMC12090506

[83] Song J Liu J Cui C Hu H Zang N Yang M . Mesenchymal stromal cells ameliorate diabetes-induced muscle atrophy through exosomes by enhancing Ampk/Ulk1-mediated autophagy. J Cachexia Sarcopenia Muscle. (2023) 14:915–29. doi: 10.1002/jcsm.13177 36708027 PMC10067482

[84] Jin J Qian F Zheng D He W Gong J He Q. Mesenchymal stem cells attenuate renal fibrosis via exosomes-mediated delivery of microrna Let-7i-5p antagomir. Int J Nanomedicine. (2021) 16:3565–78. doi: 10.2147/IJN.S299969 34079249 PMC8164705

[85] Zhang Y Le X Zheng S Zhang K He J Liu M . Microrna-146a-5p-modified human umbilical cord mesenchymal stem cells enhance protection against diabetic nephropathy in rats through facilitating M2 macrophage polarization. Stem Cell Res Ther. (2022) 13:171. doi: 10.1186/s13287-022-02855-7 35477552 PMC9044847

[86] Zhang R Tan X Zhang Y Ma Y You Z Huang Y . Punicalagin ameliorates diabetic cognitive dysfunction by inhibiting neuroinflammation via the Cx3cl1/Cx3cr1 axis. Phytother Res. (2026) 40:334–50. doi: 10.1002/ptr.70133 41308197

[87] Karaoglu IC Duymaz D Rashid MM Kizilel S. Immune-evasive beta cells in type 1 diabetes: innovations in genetic engineering, biomaterials, and computational modeling. Front Immunol. (2025) 16:1618086. doi: 10.3389/fimmu.2025.1618086 40904460 PMC12401705

[88] Lytrivi M Tong Y Virgilio E Yi X Cnop M. Diabetes mellitus and the key role of endoplasmic reticulum stress in pancreatic β cells. Nat Rev Endocrinol. (2025) 21:546–63. doi: 10.1038/s41574-025-01129-5 40467970

[89] Pan Y Shao M Li P Xu C Nie J Zhang K . Polyaminoglycoside-mediated cell reprogramming system for the treatment of diabetes mellitus. J Control Release. (2022) 343:420–33. doi: 10.1016/j.jconrel.2022.01.041 35101476

[90] Huang J Yu M Yin W Liang B Li A Li J . Development of a novel Rnai therapy: engineered Mir-31 exosomes promoted the healing of diabetic wounds. Bioact Mater. (2021) 6:2841–53. doi: 10.1016/j.bioactmat.2021.02.007 33718666 PMC7905076

[91] Basile G Qadir M Mauvais-Jarvis F Vetere A Shoba V Modell A . Emerging diabetes therapies: bringing back the β-cells. Mol Metab. (2022) 60:101477. doi: 10.1016/j.molmet.2022.101477 35331962 PMC8987999

[92] Zhou D Li S Hu G Wang Y Qi Z Xu X . Hypoglycemic effect of C. Butyricum-Pmtl007-Glp-1 engineered probiotics on type 2 diabetes mellitus. Gut Microbes. (2025) 17:2447814. doi: 10.1080/19490976.2024.2447814 39745177 PMC12931707

[93] Zhang Y Gao S Liang K Wu Z Yan X Liu W . Exendin-4 gene modification and microscaffold encapsulation promote self-persistence and antidiabetic activity of MSCs. Sci Adv. (2021) 7:eabi4379. doi: 10.1126/sciadv.abi4379 34215590 PMC11060038

[94] Ziegler A-G Cengiz E Kay TW. The future of type 1 diabetes therapy. Lancet. (2025) 406:1520–34. doi: 10.1016/S0140-6736(25)01438-2 40983070

[95] Yang R Xu H Pedersen NL Li X Yu J Bao C . A healthy lifestyle mitigates the risk of heart disease related to type 2 diabetes: a prospective nested case–control study in a nationwide Swedish twin cohort. Diabetologia. (2021) 64:530–9. doi: 10.1007/s00125-020-05324-z 33169206 PMC7864843

[96] Gonzalez-Manzanares R Anguita-Gámez M Muñiz J Barrios V Gimeno-Orna JA Pérez A . Prevalence and incidence of heart failure in type 2 diabetes patients: results from a nationwide prospective cohort—the diabet-Ic study. Cardiovasc Diabetol. (2024) 23:253. doi: 10.1186/s12933-024-02358-0 39014420 PMC11253346

[97] Iwase M Ohkuma T Fujii H Oku Y Higashi T Oshiro A . Incidence and risks of coronary heart disease and heart failure in Japanese patients with type 2 diabetes mellitus: the fukuoka diabetes registry. Diabetes Res Clin Pract. (2023) 201:110732. doi: 10.1016/j.diabres.2023.110732 37245724

[98] Seferović PM Paulus WJ Rosano G Polovina M Petrie MC Jhund PS . Diabetic myocardial disorder. A clinical consensus statement of the heart failure association of the Esc and the Esc working group on myocardial & pericardial diseases. Eur J Heart Failure. (2024) 26:1893–903. doi: 10.1002/ejhf.3347 38896048

[99] Abudureyimu M Luo X Wang X Sowers JR Wang W Ge J . Heart failure with preserved ejection fraction (Hfpef) in type 2 diabetes mellitus: from pathophysiology to therapeutics. J Mol Cell Biol. (2022) 14:mjac028. doi: 10.1093/jmcb/mjac028 35511596 PMC9465638

[100] Thakur S Chahal S Jadhav MS Mohanty P Gaikwad AB Sindhu J . Design and development of chromene-3-carboxylate derivatives as antidiabetic agents: exploring the antidiabetic potential via dual inhibition of angiotensin II type 1 receptor and neprilysin enzyme. Eur J Med Chem. (2025) 293:117705. doi: 10.1016/j.ejmech.2025.117705 40354719

[101] Ho KL Karwi QG Connolly D Pherwani S Ketema EB Ussher JR . Metabolic, structural and biochemical changes in diabetes and the development of heart failure. Diabetologia. (2022) 65:411–23. doi: 10.1007/s00125-021-05637-7 34994805

[102] Bellemare M Bourcier L Iglesies-Grau J Boulet J O'Meara E Bouabdallaoui N. Mechanisms of diabetic cardiomyopathy: focus on inflammation. Diabetes Obes Metab. (2025) 27:2326–38. doi: 10.1111/dom.16242 39930551 PMC11964996

[103] Elnwasany A Ewida HA Szweda PA Szweda LI. Inhibition of pyruvate dehydrogenase in the heart as an initiating event in the development of diabetic cardiomyopathy. Antioxidants. (2023) 12:756. doi: 10.3390/antiox12030756 36979003 PMC10045649

[104] Zhu D Zhang X Wang F Ye Q Yang C Liu D. Irisin rescues diabetic cardiac microvascular injury via Erk1/2/Nrf2/Ho-1 mediated inhibition of oxidative stress. Diabetes Res Clin Pract. (2022) 183:109170. doi: 10.1016/j.diabres.2021.109170 34863716

[105] Smati H Qadeer YK Rodriguez M Moras E Fonarow GC Isaacs SD . Diabetic cardiomyopathy: what clinicians should know. Am J Med. (2025) 138:387–95. doi: 10.1016/j.amjmed.2024.10.026 39505128

[106] Russell-Hallinan A Karuna N Lezoualc'h F Matullo G Baker H Bernard M . Established and emerging roles of epigenetic regulation in diabetic cardiomyopathy. Diabetes Metab Res Rev. (2025) 41:e70081. doi: 10.1002/dmrr.70081 40831067 PMC12365466

[107] Sabouret P Galati G Angoulvant D Germanova O Castelletti S Pathak A . The interplay between cardiology and diabetology: a renewed collaboration to optimize cardiovascular prevention and heart failure management. Eur Heart J Cardiovasc Pharmacother. (2020) 6:394–404. doi: 10.1093/ehjcvp/pvaa051 32402065

[108] Savarese G Butler J Lund LH Bhatt DL Anker SD. Cardiovascular effects of non-insulin glucose-lowering agents: a comprehensive review of trial evidence and potential cardioprotective mechanisms. Cardiovasc Res. (2022) 118:2231–52. doi: 10.1093/cvr/cvab271 34390570

[109] Giugliano D Scappaticcio L Longo M Bellastella G Esposito K. Glp-1 receptor agonists vs. Sglt-2 inhibitors: the gap seems to be leveling off. Cardiovasc Diabetol. (2021) 20:205. doi: 10.1186/s12933-021-01400-9 34641876 PMC8513211

[110] Marx N Husain M Lehrke M Verma S Sattar N. Glp-1 receptor agonists for the reduction of atherosclerotic cardiovascular risk in patients with type 2 diabetes. Circulation. (2022) 146:1882–94. doi: 10.1161/CIRCULATIONAHA.122.059595 36508493

[111] Giugliano D Longo M Signoriello S Maiorino MI Solerte B Chiodini P . The effect of Dpp-4 inhibitors, Glp-1 receptor agonists and Sglt-2 inhibitors on cardiorenal outcomes: a network meta-analysis of 23 Cvots. Cardiovasc Diabetol. (2022) 21:42. doi: 10.1186/s12933-022-01474-z 35296336 PMC8925229

[112] Zhou Y Suo W Zhang X Liang J Zhao W Wang Y . Targeting mitochondrial quality control for diabetic cardiomyopathy: therapeutic potential of hypoglycemic drugs. Biomed Pharmacother. (2023) 168:11. doi: 10.1016/j.biopha.2023.115669 37820568

[113] Neuen BL Heerspink HJ Vart P Claggett BL Fletcher RA Arnott C . Estimated lifetime cardiovascular, kidney, and mortality benefits of combination treatment with Sglt2 inhibitors, Glp-1 receptor agonists, and nonsteroidal Mra compared with conventional care in patients with type 2 diabetes and albuminuria. Circulation. (2024) 149:450–62. doi: 10.1161/CIRCULATIONAHA.123.067584 37952217

[114] Hu Q Qu C Xiao X Zhang W Jiang Y Wu Z . Flavonoids on diabetic nephropathy: advances and therapeutic opportunities. Chin Med. (2021) 16:74. doi: 10.1186/s13020-021-00485-4 34364389 PMC8349014

[115] Ke G Chen X Liao R Xu L Zhang L Zhang H . Receptor activator of Nf-κb mediates podocyte injury in diabetic nephropathy. Kidney Int. (2021) 100:377–90. doi: 10.1016/j.kint.2021.04.036 34051263

[116] Hu S Hang X Wei Y Wang H Zhang L Zhao L. Crosstalk among podocytes, glomerular endothelial cells and mesangial cells in diabetic kidney disease: an updated review. Cell Commun Signal. (2024) 22:136. doi: 10.1186/s12964-024-01502-3 38374141 PMC10875896

[117] Cansby E Caputo M Gao L Kulkarni NM Nerstedt A Ståhlman M . Depletion of protein kinase Stk25 ameliorates renal lipotoxicity and protects against diabetic kidney disease. JCI Insight. (2020) 5:e140483. doi: 10.1172/jci.insight.140483 33170807 PMC7819747

[118] Wang D Li J Luo G Zhou J Wang N Wang S . Nox4 as a novel therapeutic target for diabetic vascular complications. Redox Biol. (2023) 64:102781. doi: 10.1016/j.redox.2023.102781 37321060 PMC10363438

[119] Dai X Liao R Liu C Liu S Huang H Liu J . Epigenetic regulation of txnip-mediated oxidative stress and Nlrp3 inflammasome activation contributes to Sahh inhibition-aggravated diabetic nephropathy. Redox Biol. (2021) 45:102033. doi: 10.1016/j.redox.2021.102033 34119876 PMC8209273

[120] Hu X Chen S Ye S Chen W Zhou Y. New insights into the role of immunity and inflammation in diabetic kidney disease in the omics era. Front Immunol. (2024) 15:1342837. doi: 10.3389/fimmu.2024.1342837 38487541 PMC10937589

[121] Tuttle KR Agarwal R Alpers CE Bakris GL Brosius FC Kolkhof P . Molecular mechanisms and therapeutic targets for diabetic kidney disease. Kidney Int. (2022) 102:248–60. doi: 10.1016/j.kint.2022.05.012 35661785

[122] Giorgino F Vora J Fenici P Solini A. Renoprotection with Sglt2 inhibitors in type 2 diabetes over a spectrum of cardiovascular and renal risk. Cardiovasc Diabetol. (2020) 19:196. doi: 10.1186/s12933-020-01163-9 33222693 PMC7680601

[123] Georgianos PI Vaios V Koufakis T Liakopoulos V. Slowing the progression of chronic kidney disease in patients with type 2 diabetes using four pillars of therapy: the time to act is now. Drugs. (2024) 84:1337–46. doi: 10.1007/s40265-024-02091-8 39259460

[124] Ashfaq A Meineck M Pautz A Arioglu-Inan E Weinmann-Menke J Michel MC . Systematic review on renal effects of Sglt2 inhibitors in rodent models of diabetic nephropathy. Pharmacol Ther. (2023) 249:108503. doi: 10.1016/j.pharmthera.2023.108503 37495021

[125] Sen T Heerspink HJ. A kidney perspective on the mechanism of action of sodium glucose co-transporter 2 inhibitors. Cell Metab. (2021) 33:732–9. doi: 10.1016/j.cmet.2021.02.016 33691091

[126] Ceriello A Rodbard HW Battelino T Brosius F Cosentino F Green J . Data from network meta-analyses can inform clinical practice guidelines and decision-making in diabetes management: perspectives of the taskforce of the guideline workshop. Cardiovasc Diabetol. (2023) 22:277. doi: 10.1186/s12933-023-01993-3 37833776 PMC10576408

[127] Chen MD Deng CF Chen PF Li A Wu HZ Ouyang F . Non-invasive metabolic biomarkers in initial cognitive impairment in patients with diabetes: a systematic review and meta-analysis. Diabetes Obes Metab. (2024) 26:5519–36. doi: 10.1111/dom.15916 39233493

[128] Ríos JA Bórquez JC Godoy JA Zolezzi JM Furrianca MC Inestrosa NC. Emerging role of metformin in Alzheimer's disease: a translational view. Ageing Res Rev. (2024) 100:102439. doi: 10.1016/j.arr.2024.102439 39074563

[129] Gustafson D DiStefano PV Wang XF Wu R Ghaffari S Ching C . Circulating small extracellular vesicles mediate vascular hyperpermeability in diabetes. Diabetologia. (2024) 67:1138–54. doi: 10.1007/s00125-024-06120-9 38489029 PMC11058313

[130] Hoyos CM Colagiuri S Turner A Ireland C Naismith SL Duffy SL. Brain oxidative stress and cognitive function in older adults with diabetes and pre-diabetes who are at risk for dementia. Diabetes Res Clin Pract. (2022) 184:109178. doi: 10.1016/j.diabres.2021.109178 34958845

[131] Bailey J Coucha M Bolduc DR Burnett FN Barrett AC Ghaly M . Glp-1 receptor nitration contributes to loss of brain pericyte function in a mouse model of diabetes. Diabetologia. (2022) 65:1541–54. doi: 10.1007/s00125-022-05730-5 35687178 PMC11973880

[132] Siddeeque N Hussein MH Abdelmaksoud A Bishop J Attia AS Elshazli RM . Neuroprotective effects of Glp-1 receptor agonists in neurodegenerative disorders: a large-scale propensity-matched cohort study. Int Immunopharmacol. (2024) 143(Pt 3):113537. doi: 10.1016/j.intimp.2024.113537 39486172

[133] Tseng P-T Zeng B-Y Hsu C-W Hung C-M Carvalho AF Stubbs B . The pharmacodynamics-based prophylactic benefits of Glp-1 receptor agonists and Sglt2 inhibitors on neurodegenerative diseases: evidence from a network meta-analysis. BMC Med. (2025) 23:197. doi: 10.1186/s12916-025-04018-w 40189519 PMC11974209

[134] Nowell J Blunt E Edison P. Incretin and insulin signaling as novel therapeutic targets for Alzheimer's and Parkinson's disease. Mol Psychiatry. (2023) 28:217–29. doi: 10.1038/s41380-022-01792-4 36258018 PMC9812772

[135] de Paiva IHR da Silva RS Mendonça IP de Souza JRB Peixoto CA. Semaglutide attenuates anxious and depressive-like behaviors and reverses the cognitive impairment in a type 2 diabetes mellitus mouse model via the microbiota-gut-brain axis. J Neuroimmune Pharmacol. (2024) 19:36. doi: 10.1007/s11481-024-10142-w 39042202

[136] Roy A Dawson VL Dawson TM. From metabolism to mind: the expanding role of the Glp-1 receptor in neurotherapeutics. Neurotherapeutics. (2025) 22:e00712. doi: 10.1016/j.neurot.2025.e00712 40738791 PMC12491786

[137] Zhang P Mao C Sun A Yang Y Hou Y Fu Z . Real-world observations of Glp-1 receptor agonists and Sglt-2 inhibitors as potential treatments for Alzheimer's disease. Alzheimers Dement. (2025) 21:e70639. doi: 10.1002/alz.70639 40898408 PMC12404899

[138] Zhang Y Chen H Feng Y Liu M Lu Z Hu B . Activation of Ampk by Glp-1r agonists mitigates Alzheimer-related phenotypes in transgenic mice. Nat Aging. (2025) 5:1097–113. doi: 10.1038/s43587-025-00869-3 40394225

[139] Au HCT Lam PH Lim PK. McIntyre RS. Role of glucagon-like peptide-1 on amyloid, tau, and α-synuclein: target engagement and rationale for the development in neurodegenerative disorders. Neurosci Biobehav Rev. (2025) 173:106159. doi: 10.1016/j.neubiorev.2025.106159 40252880

[140] Lu X Xie Q Pan X Zhang R Zhang X Peng G . Type 2 diabetes mellitus in adults: pathogenesis, prevention and therapy. Signal Transduct Target Ther. (2024) 9:262. doi: 10.1038/s41392-024-01951-9 39353925 PMC11445387

[141] Ke D Zhang Z Liu J Chen P Dai Y Sun X . Ripk1 and Ripk3 inhibitors: potential weapons against inflammation to treat diabetic complications. Front Immunol. (2023) 14:1274654. doi: 10.3389/fimmu.2023.1274654 37954576 PMC10639174

[142] Sabaratnam R Skov V Paulsen SK Juhl S Kruse R Hansen T . A signature of exaggerated adipose tissue dysfunction in type 2 diabetes is linked to low plasma adiponectin and increased transcriptional activation of proteasomal degradation in muscle. Cells. (2022) 11:2005. doi: 10.3390/cells11132005 35805088 PMC9265693

[143] Faria BQ Calixto PS Picheth G Ferreira LM Rego FGM Guerra JFC . Palmitate-induced hepatic insulin resistance as an in vitro model for natural and synthetic drug screening: a scoping review of therapeutic candidates and mechanisms. Chem Biol Interact. (2025) 420:111717. doi: 10.1016/j.cbi.2025.111717 40850566

[144] Carpentier AC. Tracers and imaging of fatty acid and energy metabolism of human adipose tissues. Physiology. (2024) 39:61–72. doi: 10.1152/physiol.00012.2023 38113392 PMC11283904

[145] Kumar R Chinala A Grandhe D Endicott SJ Garcia MA Campen MJ . Metals in the human liver: an underappreciated risk factor of hepatic insulin resistance and associated pathophysiology. Environ Pollut. (2025) 383:126844. doi: 10.1016/j.envpol.2025.126844 40683376 PMC12424272

[146] Vezza T de Marañón AM Canet F Díaz-Pozo P Marti M D'Ocon P . Micrornas and oxidative stress: an intriguing crosstalk to be exploited in the management of type 2 diabetes. Antioxidants. (2021) 10:802. doi: 10.3390/antiox10050802 34069422 PMC8159096

[147] Llorián-Salvador M Cabeza-Fernández S Gomez-Sanchez JA de la Fuente AG. Glial cell alterations in diabetes-induced neurodegeneration. Cell Mol Life Sci. (2024) 81:47. doi: 10.1007/s00018-023-05024-y 38236305 PMC10796438

[148] Zhang Z He X Sun Y Li J Sun J. Type 2 diabetes mellitus: a metabolic model of accelerated aging - multi-organ mechanisms and intervention approaches. Aging Dis. (2025). doi: 10.14336/AD.2025.0233 40423634 PMC13061546

[149] Love KM Barrett EJ Malin SK Reusch JE Regensteiner JG Liu Z. Diabetes pathogenesis and management: the endothelium comes of age. J Mol Cell Biol. (2021) 13:500–12. doi: 10.1093/jmcb/mjab024 33787922 PMC8530521

[150] Caturano A Rocco M Tagliaferri G Piacevole A Nilo D Di Lorenzo G . Oxidative stress and cardiovascular complications in type 2 diabetes: from pathophysiology to lifestyle modifications. Antioxidants. (2025) 14:72. doi: 10.3390/antiox14010072 39857406 PMC11759781

[151] Xourafa G Korbmacher M Roden M. Inter-organ crosstalk during development and progression of type 2 diabetes mellitus. Nat Rev Endocrinol. (2024) 20:27–49. doi: 10.1038/s41574-023-00898-1 37845351

[152] Li J-M Li X Chan LW Hu R Zheng T Li H . Lipotoxicity-polarised macrophage-derived exosomes regulate mitochondrial fitness through Miro1-mediated mitophagy inhibition and contribute to type 2 diabetes development in mice. Diabetologia. (2023) 66:2368–86. doi: 10.1007/s00125-023-05992-7 37615690

[153] Wu Y Yang M Wu S-b Luo P-q Zhang C Ruan C-s . Zinc finger bed-type containing 3 promotes hepatic steatosis by interacting with polypyrimidine tract-binding protein 1. Diabetologia. (2024) 67:2346–66. doi: 10.1007/s00125-024-06224-2 39037604

[154] Bendixen SM Jakobsgaard PR Hansen D Hejn KH Terkelsen MK Bjerre FA . Single cell-resolved study of advanced murine mash reveals a homeostatic pericyte signaling module. J Hepatol. (2024) 80:467–81. doi: 10.1016/j.jhep.2023.11.001 37972658

[155] Kang M Song J Kang ES Jang S Kwak T Kim Y . Pathophysiology, development, and mortality of major non-communicable diseases in metabolic dysfunction-associated steatotic liver disease: a comprehensive review. Int J Biol Sci. (2025) 21:5691. doi: 10.7150/ijbs.117211 41079926 PMC12509686

[156] Mancina RM Valenti L Romeo S. Human genetics of steatotic liver disease: insights into insulin resistance and lipid metabolism. Nat Metab. (2025) 7:2199–211. doi: 10.1038/s42255-025-01394-8 41107482

[157] Mathiesen DS Lund A Holst JJ Knop FK Lutz TA Bagger JI. Therapy of endocrine disease: amylin and calcitonin–physiology and pharmacology. Eur J Endocrinol. (2022) 186:R93–111. doi: 10.1530/EJE-21-1261 35353712

[158] DeMarsilis A Reddy N Boutari C Filippaios A Sternthal E Katsiki N . Pharmacotherapy of type 2 diabetes: an update and future directions. Metabolism. (2022) 137:155332. doi: 10.1016/j.metabol.2022.155332 36240884

[159] Gunasekar SK Xie L Kumar A Hong J Chheda PR Kang C . Small molecule Swell1 complex induction improves glycemic control and nonalcoholic fatty liver disease in murine type 2 diabetes. Nat Commun. (2022) 13:784. doi: 10.1038/s41467-022-28435-0 35145074 PMC8831520

[160] Kawamura G Kokaji T Kawata K Sekine Y Suzuki Y Soga T . Optogenetic decoding of Akt2-regulated metabolic signaling pathways in skeletal muscle cells using transomics analysis. Sci Signal. (2023) 16:eabn0782. doi: 10.1126/scisignal.abn0782 36809024

[161] Vishnoi S Bhattacharya S Walsh EM Okoh GI Thompson D. Computational peptide design cotargeting glucagon and glucagon-like peptide-1 receptors. J Chem Inf Model. (2023) 63:4934–47. doi: 10.1021/acs.jcim.3c00752 37523325 PMC10428222

[162] Thai PN Miller CV King MT Schaefer S Veech RL Chiamvimonvat N . Ketone ester D-β-hydroxybutyrate-(R)-1, 3 butanediol prevents decline in cardiac function in type 2 diabetic mice. J Am Heart Assoc. (2021) 10:e020729. doi: 10.1161/JAHA.120.020729 34583524 PMC8649133

[163] Zhao Z Deng X Jia J Zhao L Wang C Cai Z . Angiopoietin-like protein 8 (betatrophin) inhibits hepatic gluconeogenesis through Pi3k/Akt signaling pathway in diabetic mice. Metabolism. (2022) 126:154921. doi: 10.1016/j.metabol.2021.154921 34715116

[164] Maccari R Wolber G Genovese M Sardelli G Talagayev V Balestri F . Designed multiple ligands for the treatment of type 2 diabetes mellitus and its complications: discovery of (5-arylidene-4-Oxo-2-thioxothiazolidin-3-Yl) alkanoic acids active as novel dual-targeted Ptp1b/Akr1b1 inhibitors. Eur J Med Chem. (2023) 252:115270. doi: 10.1016/j.ejmech.2023.115270 36934484

[165] Li JH Florez JC. On the verge of precision medicine in diabetes. Drugs. (2022) 82:1389–401. doi: 10.1007/s40265-022-01774-4 36123514 PMC9531144

[166] Cabré F Centelles JJ Cascante M. From current therapeutics to multitarget ligands: a review of diabetes pharmacological treatments. Pharmaceutics. (2025) 17:1125. doi: 10.3390/pharmaceutics17091125 41012462 PMC12473160

[167] Sridhar VS Limonte CP Groop P-H Heerspink HJ Pratley RE Rossing P . Chronic kidney disease in type 1 diabetes: translation of novel type 2 diabetes therapeutics to individuals with type 1 diabetes. Diabetologia. (2024) 67:3–18. doi: 10.1007/s00125-023-06015-1 37801140

[168] He F Su S Song R Li Y Zou L Li Z . Elucidating key components and mechanisms underlying the synergistic anti-type 2 diabetes effect of *Morus alba* L. and *Siraitia grosvenorii* combination: an integrated *in vitro* enzymology, untargeted metabolomics, and network pharmacology approach. Antioxidants. (2025) 14:1065. doi: 10.3390/antiox14091065 41008970 PMC12466828

[169] Mobeen B Shah M Rehman HM Jan MS Rashid U. Discovery of the selective and nanomolar inhibitor of Dpp-4 more potent than sitagliptin by structure-guided rational design. Eur J Med Chem. (2024) 279:116834. doi: 10.1016/j.ejmech.2024.116834 39265251

[170] Bonnefond A Florez JC Loos RJ Froguel P. Dissection of type 2 diabetes: a genetic perspective. Lancet Diabetes Endocrinol. (2025) 13:149–64. doi: 10.1016/S2213-8587(24)00339-5 39818223

[171] Dyson NJ Kattner N Al-Selwi Y Honkanen-Scott M Shaw MF Brack CA . Quantitative analysis of human adult pancreatic histology reveals separate fatty and fibrotic phenotypes in type 2 diabetes. Diabetologia. (2025) 68:2840-53. doi: 10.1007/s00125-025-06547-8 40991018 PMC12594667

[172] Chen K Wang H Yang Y Tang C Sun X Zhou J . Common mechanisms of gut microbe-based strategies for the treatment of intestine-related diseases: based on multi-target interactions with the intestinal barrier. Cell Commun Signal. (2025) 23:288. doi: 10.1186/s12964-025-02299-5 40528179 PMC12175372

[173] de Oliveira TL Lincevicius GS Shimoura CG Simoes-Sato AY Garcia ML Bergamaschi CT . Effects of renal denervation on cardiovascular, metabolic and renal functions in streptozotocin-induced diabetic rats. Life Sci. (2021) 278:119534. doi: 10.1016/j.lfs.2021.119534 33933461

[174] Young JB Eknoyan G. Cardiorenal syndrome: an evolutionary appraisal. Circ Heart Fail. (2024) 17:e011510. doi: 10.1161/CIRCHEARTFAILURE.123.011510 38757274

[175] Mentz RJ Brunton SA Rangaswami J. Sodium-glucose cotransporter-2 inhibition for heart failure with preserved ejection fraction and chronic kidney disease with or without type 2 diabetes mellitus: a narrative review. Cardiovasc Diabetol. (2023) 22:316. doi: 10.1186/s12933-023-02023-y 37974185 PMC10655322

[176] Demir S Nawroth PP Herzig S Ekim Üstünel B. Emerging targets in type 2 diabetes and diabetic complications. Adv Sci. (2021) 8:2100275. doi: 10.1002/advs.202100275 34319011 PMC8456215

[177] Kogot-Levin A Riahi Y Abramovich I Mosenzon O Agranovich B Kadosh L . Mapping the metabolic reprogramming induced by sodium-glucose cotransporter 2 inhibition. JCI Insight. (2023) 8:e164296. doi: 10.1172/jci.insight.164296 36809274 PMC10132155

[178] Paniagua G Couce-Sánchez M González-Blanco L Sabater C García-Fernández A Rodríguez-Revuelta J . Comparative analysis of gut microbiome-derived short-chain fatty acids in patients with severe mental disorder: insights from schizophrenia and bipolar disorder. Prog Neuropsychopharmacol Biol Psychiatry. (2025) 138:111345. doi: 10.1016/j.pnpbp.2025.111345 40147807

[179] Bai Y Zhao Y Jin J Ye Z Fan H Zhao D . Jiang tang san hao formula exerts its anti-diabetic effect by affecting the gut-microbiota-brain axis. Phytomedicine. (2024) 135:156100. doi: 10.1016/j.phymed.2024.156100 39388919

[180] Nauck MA Müller TD. Incretin hormones and type 2 diabetes. Diabetologia. (2023) 66:1780–95. doi: 10.1007/s00125-023-05956-x 37430117 PMC10474001

[181] Sbriscia M Colombaretti D Giuliani A Di Valerio S Scisciola L Rusanova I . Triglyceride glucose index predicts long-term mortality and major adverse cardiovascular events in patients with type 2 diabetes. Cardiovasc Diabetol. (2025) 24:115. doi: 10.1186/s12933-025-02671-2 40065340 PMC11895143

[182] Chen Q Xiong S Zhang Z Yu X Chen Y Ye T . Triglyceride-glucose index is associated with recurrent revascularization in patients with type 2 diabetes mellitus after percutaneous coronary intervention. Cardiovasc Diabetol. (2023) 22:284. doi: 10.1186/s12933-023-02011-2 37865753 PMC10590524

[183] Zhang J Zhan Q Deng Z Lin L Feng Z He H . Does diabetes modify the triglyceride–glucose index associated with cardiovascular events and mortality? A meta-analysis of 50 cohorts involving 7,239,790 participants. Cardiovasc Diabetol. (2025) 24:42. doi: 10.1186/s12933-025-02585-z 39871273 PMC11773825

[184] Oh R Kim S Park SH Jang M Cho SH Kim JY . Elevated triglyceride-glucose index is a risk factor for cardiovascular events in adults with type 1 diabetes: a cohort study. Cardiovasc Diabetol. (2025) 24:150. doi: 10.1186/s12933-025-02712-w 40176060 PMC11966936

[185] Wang X Zheng K Hu X Pei J. The impact of sex-related disparities on the association between triglyceride-glucose index and renal function decline in patients with type 2 diabetes: insights from the accord trial. Diabetes Res Clin Pract. (2025) 224:112163. doi: 10.1016/j.diabres.2025.112163 40250809

